# Isolation of Environmental Bacteria from Surface and Drinking Water in Mafikeng, South Africa, and Characterization Using Their Antibiotic Resistance Profiles

**DOI:** 10.1155/2014/371208

**Published:** 2014-07-06

**Authors:** Suma George Mulamattathil, Carlos Bezuidenhout, Moses Mbewe, Collins Njie Ateba

**Affiliations:** ^1^Department of Water and Sanitation, University of Limpopo, Turfloop Campus, Private Bag X 1106, Sovenga 0727, South Africa; ^2^School of Biological Science, North-West University, Potchefstroom Campus, Private Bag X 6001, Potchefstroom 2520, South Africa; ^3^Department of Biological Sciences, School of Environmental and Health Sciences, North-West University, Mafikeng Campus, Private Bag X2046, Mmabatho 2735, South Africa

## Abstract

The aim of this study was to isolate and identify environmental bacteria from various raw water sources as well as the drinking water distributions system in Mafikeng, South Africa, and to determine their antibiotic resistance profiles. Water samples from five different sites (raw and drinking water) were analysed for the presence of faecal indicator bacteria as well as* Aeromonas* and* Pseudomonas *species. Faecal and total coliforms were detected in summer in the treated water samples from the Modimola dam and in the mixed water samples, with* Pseudomonas* spp. being the most prevalent organism. The most prevalent multiple antibiotic resistance phenotype observed was KF-AP-C-E-OT-K-TM-A. All organisms tested were resistant to erythromycin, trimethoprim, and amoxicillin. All isolates were susceptible to ciprofloxacin and faecal coliforms and* Pseudomonas* spp. to neomycin and streptomycin. Cluster analysis based on inhibition zone diameter data suggests that the isolates had similar chemical exposure histories. Isolates were identified using* gyrB*,* toxA, ecfX, aerA,* and* hylH *gene fragments and* gyrB*,* ecfX, *and* hylH *fragments were amplified. These results demonstrate that (i) the drinking water from Mafikeng contains various bacterial species and at times faecal and total coliforms. (ii) The various bacteria are resistant to various classes of antibiotics.

## 1. Introduction

Water is considered a vehicle for the propagation and dissemination of human associated bacteria [1]. Safe drinking water is a fundamental human right and if contaminated with opportunistic pathogenic environmental bacteria, it may have health implications for consumers [2, 3]. Human health should therefore be protected by preventing microbial contamination of water that is intended for consumption [4]. In rural communities, untreated surface water from rivers, dams, and streams is directly used for drinking and other domestic purposes [5]. These unprotected water sources can be contaminated with microbes through rainfall run-off and agricultural inputs, mixing with sewage effluents and faeces from wild life [6, 7], which render them unacceptable for human consumption. Faecal coliforms,* Aeromonas* and* Pseudomonas,* are used as indicators of faecal contamination in water [8] and the presence of these pathogens may have severe health implications on consumers especially those that are immunocompromised [5, 9, 10].

South Africa is a semiarid country with very low rainfall and high evaporation [11] and it has a scarcity of fresh water systems due to the highly variable and spatial distribution of rainfall [12]. Moreover, safe drinking water is frequently used for unsustainable nondrinking applications such as irrigation, toilet and urinary flushing [12], and general cleaning. To manage the existing water resources and to address some of the challenges associated with water shortages in South Africa and the world at large, waste water reuse can form an important component of water demand management [13], but this waste water reuse may affect the quality of drinking water if proper treatment procedures are not implemented. Whereas waste water reuse has been extensively implemented in some European and African countries [13], yet in South Africa, only a few waste water reuse schemes have been documented [14, 15] and there is limited implementation of this alternative in communities.

Alarming increases in the consumption of antibiotics through human therapy and agricultural processes have been reported [16, 17] and this extensive usage in both human and animal medicine has resulted in the development of antibiotic-resistant bacteria which affect the treatment of infections [18, 19]. Antibiotic resistance has therefore become a major public health issue [20] and its presence in waste water, surface water, and drinking water is well documented [19–22]. The hazard associated with the pathogenicity of microbes is aggravated by its ability to resist destruction by antibiotics. Biological treatment processes in the waste water treatment plants may result in a selective increase of antibiotic-resistant bacteria and therefore increase the occurrence of multidrug-resistant organisms [23]. Although microorganisms in drinking water are reduced by chlorination, they may survive the treatment process and enter the distribution system [1]. Moreover, the presence of antibiotic resistance in microorganisms has been previously reported [24–26]. Considering the fact that the public health of a community may be related to the quality of treated waste water supplied and that public health can be protected by reducing the pathogenic microorganisms in drinking water, the present study was designed to isolate environmental bacteria from surface and drinking water in Mafikeng and identify the* Pseudomonas* and* Aeromonas* species using polymerase chain reaction (PCR). A further objective was to characterize the isolates using their antibiotic resistance profiles.

## 2. Materials and Methods

### 2.1. Study Area

Water samples were collected from five sampling points around Mafikeng, namely, both raw and treated (drinking) water from a karstic groundwater source, the Molopo Eye, both raw water and treated (drinking) water from the Modimola dam, and finally mixed water, treated water from both sources mixed in the Signal hill reservoir and distributed to some areas in the city. These sampling points were chosen for the study because water from Molopo eye and Modimola dam, after purification, is used for human consumption, and for recreational, agricultural, and industrial purposes. As few small scale of farmers live near these two water resources and the Modimola Dam receives treated sewage effluent from the Mmabatho sewage treatment plant, which is the major source of pathogens, it is therefore important to investigate the microbiological quality of water at these points.

### 2.2. Sampling

Raw and treated water samples were collected during a one-year period, in February, April, July, and October, to cover the four different seasons. Water samples from the Molopo eye and Modimola dam were collected aseptically in sterile 500 mL Duran Schott glass bottles from different sampling points by directly dipping the bottles into the surface of the water. Purified water samples were collected directly in to the sterile bottles from the tap, after letting the tap run for a minute. The samples were labelled properly and transported on ice to the laboratory for analysis. Aliquots of the samples were used for selective isolation of faecal coliforms, total coliforms,* Aeromonas*,* Pseudomonas,* and heterotrophic bacteria based on standard microbiological procedures [27].

### 2.3. Isolation, Purification, and Characterization of Planktonic Bacteria

#### 2.3.1. Isolation by Membrane Filtration

For all the samples, three volumes of 100 mL were filtered through 0.45 *μ*m pore-sized filter (cellulose nitrate membranes, Whatman Laboratory Division, Maidstone, England) using a water pump (model Sartorius 16824). These membranes were aseptically placed up on plates with appropriate selective media ensuring that no air bubbles were trapped. The selective media used are as follows. mFC agar used as a selective medium for faecal coliforms, mEndo for total coliforms, nutrient agar for heterotrophic bacteria, and Aeromonas selective medium for* Aeromonas* and* Pseudomonas* (Biolab, Merck, South Africa). All the media were prepared according to the manufacturers' instructions (Biolab, Merck, South Africa). Each sample was analysed in triplicate. In order to isolate heterotrophic bacteria, 1 mL of the treated water samples was spread onto the nutrient agar plates. Water samples from Modimola dam and Molopo eye were serially diluted and 1 mL of the 5 fold serial dilutions was spread on to the nutrient agar plates.

The plates were incubated at 37°C except for mFC agar which were incubated at 45°C for 24 hours. The colonies were enumerated, characterized, and recorded. The results were expressed as the number of faecal coliforms, total coliforms,* Pseudomonas* and* Aeromonas* in 100 mL of water, and heterotrophic bacteria in 1 mL of water. Blue colonies from mFC agar (presumptive coliforms), metallic-sheen colonies from mEndo agar (presumptive total coliforms), and yellow (presumptive* Aeromonas*) and green colonies (presumptive* Pseudomonas*) from Aeromonas selective media were picked for further work (Biolab Catalogue).

#### 2.3.2. Purification of Colonies

Colonies were purified by twice subculturing using the streaking plate method. Young cultures were used for Gram staining and all isolates were identified as Gram-negative Bacilli. All the Gram-negative isolates were subjected to primary and secondary biochemical identification tests. The bacteria that were picked to create antibiograms were streaked on to nutrient agar slants to make sample cultures and for PCR purpose.

### 2.4. Antimicrobial Susceptibility Testing

An antibiotic susceptibility test was performed using the Kirby-Bauer disk diffusion method [28]. The following antibiotic discs (Mast Diagnostics, UK) at the final concentrations that are indicated were used: ampicillin (AP) −10 *μ*g, cephalothin (KF) 5 *μ*g, streptomycin (S) 10 *μ*g, erythromycin (E) 15 *μ*g, chloramphenicol (C) 30 *μ*g, neomycin (NE) 30 *μ*g, amoxycillin (A) 10 *μ*g, ciprofloxacin (CIP) 5 *μ*g, trimethoprim (TM) 25 *μ*g, kanamycin (K) 30 *μ*g, and oxytetracycline (OT) 30 *μ*g. These antibiotics were chosen because they are either used in both human medicine and animal veterinary practice or because previous studies have reported microbial resistance to them [29].

Three colonies were picked from each sample and each colony was transferred in to 3 mL of sterile distilled water to prepare bacterial suspension. Aliquots of 100 *μ*L from each suspension were spread-plated on Mueller-Hinton agar plates. Antibiotic discs were applied on to the plates using sterile needles and the plates were incubated at 37°C for 24 hours [30]. After incubation, the antibiotic inhibition zone diameters (IZD) were measured. Results obtained were used to classify isolates as being resistant, intermediate resistant, or susceptible to a particular antibiotic using standard reference values according to National Committee for Clinical Laboratory Standards [30], now Clinical and Laboratory Standards Institute (CLSI). Multiple antibiotic resistance (MAR) phenotypes were generated for isolates that showed resistance to 3 or more antibiotics.

### 2.5. Primary Identification Tests

#### 2.5.1. Triple Sugar Iron (TSI) Test

Triple sugar iron (TSI) agar is a differential medium obtained from Biolab, Merck (South Africa), with the substrates glucose, sucrose, and lactose at sample concentrations of 0.1, 1.0, and 1.0%, respectively. It can distinguish between a number of Gram-negative enteric bacteria based on their physiological ability (or lack thereof) to metabolize lactose and/or sucrose, conduct fermentation to produce acid, produce gas during fermentation, and generate H_2_S. The media were prepared according to the manufacturer's instructions. Aliquots were placed in test tubes and autoclaved. Once out of the autoclave, the tubes were placed on a rack and clamped so that the tubes (with liquid medium in them) have a 3 cm slant with a 2 to 3 cm butt. They were allowed to cool down until they become solid. The slants are inoculated with a pure culture by streaking over the entire surface of the slant (zigzagging to cover the surface) and then stabbing deep into the butt. Incubation was at 37°C for 24 hours. If only glucose is fermented, acid is produced in the butt and it will turn yellow. However, if either sucrose or lactose is fermented, sufficient fermentation products will be formed to turn both the butt and the slant yellow. If gas is formed during the fermentation, it will be shown in the butt either as bubbles or as cracking of the agar. If no fermentation occurs, the slant and butt will remain red. The medium also contains ferrous sulphate. If the bacterium forms H_2_S, this chemical will react with the iron to form ferrous sulphide, which is seen as a black precipitate in the butt (a black butt).

#### 2.5.2. Oxidase Test

This test was performed using the test oxidase reagent (PL.390) from Mast Diagnostics (Nesto, Wirral, UK) in accordance with the manufacturer's published protocol. A well-isolated pure colony was placed on a filter paper using a sterile wire loop. A drop of test oxidase reagent was added on to it and mixed. After 30 seconds, the filter was observed for a colour change with oxidase positive isolates producing a purple colour being taken as presumptive* Aeromonas* and* Pseudomonas* isolates. Oxidase negative colonies were colourless and were presumptively considered to be* E. coli*.

### 2.6. Secondary Identification Tests

#### 2.6.1. Analytical Profile Index (API) 20E Test

The API 20E test was performed in accordance with the manufacturer's protocol (BioMérieux, 69280, Marcy I'Etoile, France) and the organisms were identified to species level using API software.

#### 2.6.2. Haemolysis on Blood Agar

Haemolysis, on blood agar (Biolab, Merck, SA) supplemented with 5% (v/v) sheep blood, was determined after the incubation of the plates at 37°C for 24 hours. Haemolysis is determined by streaking for isolation on a blood agar plate. After incubation overnight, the medium is inspected for signs of alpha- or beta-haemolysis. If the medium is discoloured or darkened after growth, the organism has demonstrated alpha-haemolysis. If the medium develops clear halo under growth, the organism is beta-haemolytic. No discernible change in the colour of the medium constitutes gamma haemolysis.

### 2.7. Extraction of Genomic DNA and PCR for the Identification of Culture Species

DNA from the isolates was extracted using the peqGOLD (PEQLAB Biotechnologie GmbH 12-3450) bacterial DNA extraction kit according to the manufacturer's protocol. The concentration of the extracted DNA in solution was determined spectrophotometrically (NanoDrop ND 1000, Thermo Scientific, USA). The integrity of the purified template DNA was assessed by conventional 1% (w/v) agarose gel.

#### 2.7.1. Identification of the Isolates by PCR Assays

The identities of the presumptive* Pseudomonas* were confirmed through amplicons of* gyrB* 222 [31],* toxA 367* [32], and* ecfX 528* [33] gene fragments, respectively. The identity of* Aeromonas* species was determined by screening them for the presence of specific virulent genes aerolysin (*aerA*) [34] and haemolysin (*hylH*) [35].

PCRs were performed using oligonucleotide primer combinations and cycling conditions that appear in Tables 1 and 2. Amplifications were performed using a Peltier Thermal Cycler (model-PTC-220DYAD DNA ENGINE, MJ Research Inc., USA). The reactions were prepared in 25 *μ*L volumes that constituted 1 *μ*g/*μ*L of the template DNA, 50 pmol of each oligonucleotide primer set, 1x PCR master mix, and RNase free water. All PCR reagents used were Fermentas, USA, products supplied by Inqaba Biotechnological Industries Pty Ltd., Pretoria, South Africa. All PCR products were stored at 4°C until used for further analysis.

#### 2.7.2. Electrophoresis of PCR Products

PCR products were separated by electrophoresis on 2% (w/v) agarose gel. Electrophoresis was conducted in a horizontal Pharmacia Biotech equipment system (model Hoefer HE 99X; Amersham Pharmacia Biotech, Sweden) for 2 h at 60 V using 1x TAE buffer (40 mM Tris, 1 mM EDTA, and 20 mM glacial acetic acid, pH 8.0). Each gel contained a 100 bp DNA molecular weight marker (Fermentas, USA). The gel was stained in ethidium bromide (0.1 *μ*g/mL) for 15 min and amplicons were visualized under UV light. A Gene Genius Bioimaging system (Syngene, Synoptics, UK) was used to capture the image using GeneSnap (version 3.07.01) software (Syngene, Synoptics, UK).

### 2.8. Statistical Analysis

Cluster analysis based on the antibiotic inhibition zone diameter data of different organisms isolated from different sites was determined using Wards algorithm and Euclidean distance of Statistica version 7.

## 3. Results

### 3.1. Occurrence of Coliform Bacteria and* Aeromonas *and* Pseudomonas* Species in Water

The primary aim of this study was to determine the levels of environmental bacteria from source and drinking water from Mafikeng. Raw water from Modimola dam and Molopo eye, treated water from these two sites, and mixed water were analysed for the presence of total coliforms, faecal coliforms, heterotrophic bacteria, and* Aeromonas* and* Pseudomonas* species.  Table 3 shows the average number of different organisms isolated from the various sites during summer and winter. Heterotrophic bacteria were isolated from all the sampling sites in both seasons and their occurrence was high. Moreover,* Pseudomonas* species, faecal and total coliforms were the most prevalent during all the seasons in both raw and treated water from Modimola dam. However,* Aeromonas* species were isolated only from the raw water samples, not from treated water from all the sites. The numbers of the different organisms isolated were higher in summer than in winter.

No total coliforms were observed in treated water from the Molopo eye.* Pseudomonas* species were isolated in large numbers from Modimola dam both in raw and treated water from this site.

### 3.2. Biochemical Tests Used to Identify the Isolates

API tests results are listed in Table 4. Various species were isolated from the water sources particularly from the untreated water. The diversity of bacteria from this source was greater than from the treated water.* Pseudomonas* spp. were isolated from the Modimola dam (untreated as well as treated) water, the Molopo eye (untreated only), and the mixed water.* Aeromonas* spp. were isolated from the untreated as well as the mixed water.* Aeromonas* and* Pseudomonas* species isolated in this study were *β*-haemolytic and indicated potential pathogenicity.

### 3.3. Antibiotic-Resistant Data of Different Isolates from Drinking Water

Isolates were subjected to an antibiotic susceptibility test using 11 different antibiotics from which their antibiotic resistance profiles and multiple antibiotic resistance phenotypes were compiled. The results obtained are depicted in Tables 5, 6, 7, and 8. The results revealed that a large proportion of the environmental isolates were resistant to erythromycin, followed by trimethoprim and amoxicillin. None of the isolates were resistant to ciprofloxacin and only very few isolates from Modimola dam were resistant to streptomycin and neomycin. Despite the fact that all isolates from Molopo eye, treated water from Molopo eye and Modimola dam, and mixed water were susceptible to neomycin, only a small proportion (20%) from Modimola dam was resistant to this antibiotic. Multiple antibiotic-resistant (MAR - 3 to 8 antibiotics) bacteria were common among the isolates.

Faecal coliforms from all sites were susceptible to streptomycin, neomycin, and ciprofloxacin, respectively. The highest number of bacteria was resistant to erythromycin. However, two isolates from Modimola dam were susceptible to erythromycin. Heterotrophic bacteria were isolated from all sites and were also resistant to several antibiotics. Isolates from all sites were susceptible to streptomycin, neomycin, and ciprofloxacin.* Aeromonas* species isolated were all resistant to erythromycin. All of these species were susceptible to ciprofloxacin. However, isolates from Molopo eye were susceptible to kanamycin, neomycin, and streptomycin while isolates from the Modimola dam were resistant to most antibiotics. All these isolates were resistant to ampicillin, oxytetracycline, trimethoprim, and amoxicillin. There were single isolates from* Pseudomonas* spp. for Modimola dam, Molopo eye (untreated water sources), and the Modimola dam treated water and mixed treated water. All the isolates were resistant to up to eight antibiotics but all were susceptible to streptomycin, neomycin, and ciprofloxacin.

#### 3.3.1. Predominant Multiple Antibiotic-Resistant (MAR) Phenotypes Observed among the Bacterial Groups

Different types of multiple antibiotic resistance patterns were observed amongst all the bacterial groups isolated from the various sites. In some cases, some of the isolates were resistance to up to ten different antibiotics. The predominant antibiotic-resistant phenotypes that were obtained for the groups from different sites are depicted in Table 9. Similar types of MAR phenotypes were observed in isolates from groups.

### 3.4. Cluster Analysis of the Isolates Based on Inhibition Zone Diameter Data

All the* Pseudomonas*,* Aeromonas,* and heterotrophic bacterial isolates were subjected to cluster analysis based on their inhibition zone diameter (IZD) data and a dendrogram was generated using Ward's method. This approach was used as a tool in determining the commonness and in resolving differences between the MAR phenotypes of different isolates.

**Figure 1:**
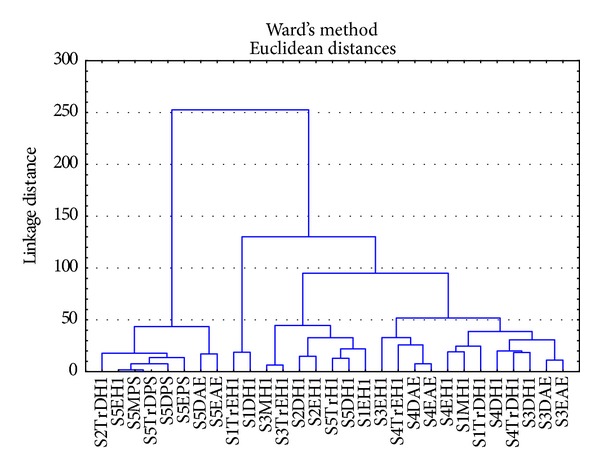
Cluster analysis for* Pseudomonas*,* Aeromonas,* and heterotrophic bacteria isolated from different sites within the various clusters (PS:* Pseudomonas*; AE:* Aeromonas*, H: heterotrophic bacteria).

*Pseudomonas*,* Aeromonas,* and heterotrophic bacteria isolated from all sites were subjected to cluster analysis and two main clusters were observed (Figure 1) with the number of isolates from different sites within the clusters being depicted in Table 10. Both clusters have subclusters. Cluster 1 is a large cluster and has a total of 21* Aeromonas* and heterotrophic bacterial isolates, while cluster 2 is a mixed cluster with* Pseudomonas*,* Aeromonas,* and heterotrophic bacteria.* Pseudomonas* is only found in cluster 2.

The cluster analysis did not demonstrate groupings based on bacterial groups or sites. This suggests that all the isolates had similar chemical exposure histories.

### 3.5. Molecular Identification of* Pseudomonas* and* Aeromonas* Species

**Figure 2:**
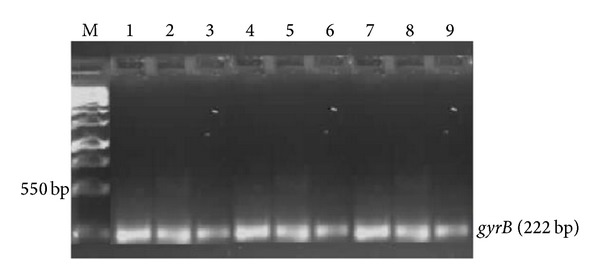
Image of a composite agarose (1% w/v) gel depicting DNA extracted from* Pseudomonas* species. Lane M (1 kb DNA Ladder); Lane 1–9 (*gyrB* gene fragments (222 bp) from* Pseudomonas* species isolated from different sites.

**Figure 3:**
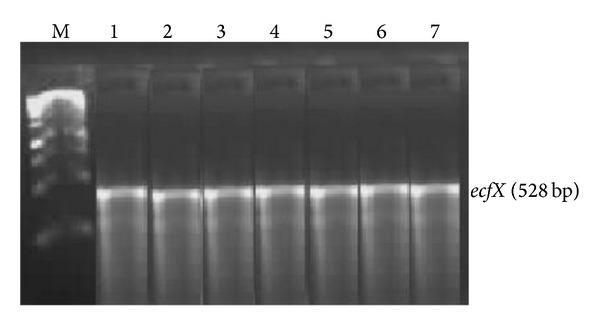
Image of a composite agarose (1% w/v) gel depicting genomic DNA extracted from* Pseudomonas* species. Lane M (1 kb DNA Ladder); Lane 1–7* ecfX* gene fragments from* Pseudomonas* species isolated from different sites.

Specific PCR was used to determine the identities of presumptive* Pseudomonas* species through amplification of the* gyrB*,* toxA,* and the* ecfX* gene fragments. Figures 2 and 3 show agarose (1% w/v) gels indicating* gyrB* and* ecfX* gene fragments generated by PCR using genomic DNA extracted from* Pseudomonas* species isolated from different sites. Gel electrophoresis of PCR products revealed the desired 222 bp and 528 bp fragments for the* gyrB* and* ecfX* gene fragments, respectively. A total of 61 isolates were screened and 17 were positively identified.

**Figure 4:**
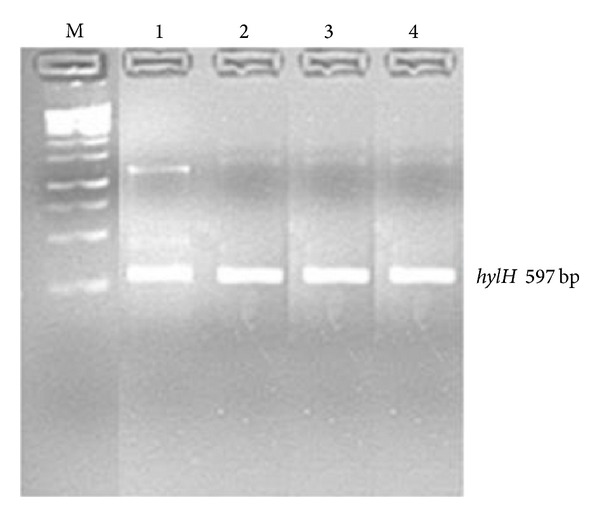
Image of a composite agarose (1% w/v) gel depicting the* hylH* gene from* Aeromonas* species. Lane M (1 kb DNA Ladder); Lanes 1–4 (*hylH* gene fragments from* Aeromonas* species isolated from different sites).

The identity of* Aeromonas* species was determined by screening them for the presence of specific virulent genes aerolysin (*aerA*) [34] and haemolysin (*hylH*) [35].  Figure 4 shows agarose (1% w/v) gels indicating* hylH* gene fragments generated by PCR using genomic DNA extracted from* Aeromonas* species isolated from different sites.

PCR results depicted in Figures 2–4 support the biochemical identification of the* Pseudomonas* spp. and* Aeromonas* spp. Furthermore, it demonstrated that certain virulence genes were present in these isolates. This is also supported by positive *β*-haemolytic activity indicating the pathogenic potential of these environmental isolated species.

## 4. Discussion

One of the objectives of this study was to isolate environmental bacteria from surface and drinking water in Mafikeng, North West Province, South Africa. A motivation for this study was the numerous reports about the occurrence of pathogenic microorganisms in drinking water and the associated diseases [6, 36–40]. In addition to this, resistance of microorganisms to antibiotics of clinical interest has previously been reported in the area [24, 26, 29]. The study demonstrated the occurrence of total coliforms, faecal coliforms, heterotrophic bacteria, and* Aeromonas* and* Pseudomonas* in water samples analysed which indicated the incidence of water contamination as some of these species are indicators of faecal contamination [8]. These organisms may harbour potential pathogens and the presence of pathogenic organisms that can pose severe health risks to consumers in general and immunocompromised individuals in particular [5]. Reduction in the number of bacteria in the treated water could be due to the treatment process, when comparing drinking water to raw water. However, occurrence of bacteria in the water after treatment could also harbour potential pathogens and the health risk caused by these should be taken into consideration when water is distributed. This is of particular importance when the drinking water abstraction and purification facility are at a relatively short distance from the sewage treatment and effluent disposal facility. In Mafikeng, the latter is the case. One of the waste water treatment plants in Mafikeng is situated on the Modimola dam and is upstream from the drinking water purification plant. This may explain the larger number and diversity of isolates from this water. Results from this and other studies show that drinking water produced from the Modimola dam should be further analysed and that general microbiological tests that only include faecal coliforms and* E. coli* may be insufficient.

Molopo eye is a protected spring into which no treated sewage effluent is being discharged. However, there is housing development that is linked to septic tanks. The occurrence of bacteria at this site may be due to contamination from human activity (septic tank, diving), pets, and wild birds. The levels of bacteria from this site were lower and after treatment very low numbers of heterotrophic bacteria and no* Aeromonas* and* Pseudomonas* spp. were detected. However, the overall results obtained in the present study indicated that the organisms in treated water may have survived the treatment process. When the levels of* Pseudomonas* spp. in the raw water were high, this increased the chances of finding this species in treated water.

A further objective of this study was to characterise the isolates using their antibiotic resistance profiles. The results revealed that a large proportion of the environmental isolates were resistant to erythromycin followed by trimethoprim and amoxicillin. The trend was in accordance with earlier studies that showed resistance towards *β*-lactam, macrolides, and phenicols [41, 42]. All the isolates from all the sites were resistant to erythromycin which was in accordance with the study conducted by [20] where most isolates were also resistant to erythromycin and tetracycline (92.9%). A large percentage (60%–100%) of isolates were also resistant to trimethoprim and ampicillin. All these results could be attributed to the overuse of these antibiotics in the clinical and veterinary setting. All isolates were found to be susceptible to streptomycin and ciprofloxacin in line with an earlier observation [43]. This is not in agreement with yet another observation [44] where increased resistance against ciprofloxacin in* E. coli* and* Pseudomonas* was observed.

*Coliforms* detected in all samples in the present study were resistant to 3 or more classes of antibiotics. This is supported by previous studies which obtained similar results [19, 20]. Multiple antibiotic resistance (MAR) in* E. coli* and* Pseudomonas* spp. isolated from environmental samples and hospitalized patients had previously been reported [45–49]. These studies support the findings of the present study. Antibiotic resistance patterns were generally similar in all different sites. However, the largest numbers of isolates that were resistant to the largest numbers of antibiotics were from the Modimola dam.

Although heterotrophic bacteria were isolated from all sites and exhibited antibiotic resistance, isolates from all sites were susceptible to streptomycin, neomycin, and ciprofloxacin which agree with the susceptibility to ciprofloxacin observed by Biyela et al. [5]. They found the highest resistant towards trimethoprim followed by *β*-lactams and tetracycline. This was also similar to the results by Biyela et al. [5]. All isolates from treated water from Molopo eye and mixed water were sensitive to erythromycin.

High loads of heterotrophic bacteria and opportunistic pathogens that harbour multiple drug resistance determinants pose significant health hazards to consumers, especially those whose immune systems are compromised [50]. Associated virulence factors give heterotrophic bacteria the ability to act as opportunistic pathogens [10]. These bacteria (heterotrophic bacteria) are generally harmless; but some may harbour pathogenic features which may cause potential health risks to humans and animals. Thus, the concern that we report here is the high levels of heterotrophic bacteria from water sources, particularly drinking water from Mafikeng. What is also of concern are the multiple antibiotic resistance patterns observed amongst these isolates. This may be a therapeutic challenge if individuals infected by these opportunistic pathogens are to be treated by available antibiotics.

In a study conducted by Sharma et al. [7] all* Aeromonas* spp. isolated from fresh water were resistant to ampicillin. However, in this study resistance to tetracycline, trimethoprim, and cephalothin was observed. Furthermore, resistance of* Aeromonas* and* Pseudomonas* spp. have also been reported among isolates from municipally treated tap and raw water samples [51, 52]. These findings support the observations in the present study that shows resistance of* Aeromonas* and* Pseudomonas* spp. to a variety of antibiotics.

Wide distribution of antibiotic-resistant bacteria in surface and ground waters has been reported in previous studies [53, 54] and results of the present study is thus not uncommon. There is, however, concern about the increase in the incidence of MAR in organisms from various sources [55]. Resistance could be attributed to heavy contamination from sewage effluent, surface runoff, agricultural activities, wild life, industrial pollution, and so forth. Considering the high incidence of HIV/AIDS in South Africa, the importance of such findings cannot be overemphasized. The results of this study on bacterial resistance profiles are consistent with previous studies in other surface and drinking water systems [10, 20, 55].

Besides receiving treated sewage effluent, Modimola dam also supports small scale farming communities close to the dam. Livestock graze around the dam and their faeces is washed off into the dam during the rainy season. Therefore, the dam could serve as a reservoir of antibiotic-resistant bacteria with sewage contamination contributing to the dissemination of antibiotic-resistant bacteria in this environment. There may thus be a link between the resistant isolates from sewage treatment plant, Modimola dam, and the treated water. This aspect, however, needs to be demonstrated which could be done in follow-up studies. Cluster analysis indicated that isolates from all sites had similar antibiotic resistance profiles. This data indicates common chemical exposure histories of the various isolates. Such an approach could be used to investigate the relationship between sewage treatment plant, Modimola dam, and treated water. The results from the present study show that bacterial isolates from the Modimola dam and treated water had similar antibiotic profiles.

Another objective of this study was to identify the* Pseudomonas* and* Aeromonas* species using PCR. Isolates were identified using* gyrB*,* ecfX,* and* hylH*. The* ecfX* gene encodes an extra cytoplasmic function sigma factor, which may be involved in haem uptake or virulence [56], whereas the* gyrB* gene encodes the DNA gyrase subunit B, a protein which plays a crucial role in the DNA replication process [34] and* toxA* encoding the exotoxin A precursor [33]. PCR assay targeting the* ecfX* and* gyrB* genes is suitable for the identification of* P. aeruginosa* [56]. In this study, biochemical identification methods were supported by PCR assays to identify the* Pseudomonas* spp. isolated.

Application of PCR technique to target* gyrB, aerA,* and* hylH* genes are molecular markers for identifying and screening potentially virulent* Aeromonas* species in food and the environment [34, 35]. In this study, this approach supported biochemical data to identify the various* Aeromonas* spp. The detection of* Aeromonas* species in the present study that harbour putative virulence factors and resistance to various antibiotics indicates that drinking water that is supplied to the community could serve as a source for the transmission of pathogens to humans [51, 57].

Overall, the results from the present highlight the need to conduct studies to determine the prevalence of antibiotic-resistant genes among environmental isolates and the dissemination of resistant genes among the pathogens. This would provide information about the health risks associated with the consumption of contaminated water.

## 5. Conclusions

An evaluation of the bacteriological quality of drinking water in the present study confirmed the presence of various bacterial species including opportunistic pathogens such as* Aeromonas* and* Pseudomonas* spp. These organisms were resistant to several classes of antibiotics. Undesirable properties of water quality caused by the presence of drug-resistant bacteria can pose a negative impact on human health.

The data on multiple antibiotic resistance (MAR) profiles of bacterial isolates from water and the resistance patterns of organisms in drinking water in Mafikeng suggested that there has been an indiscriminate use of the antibiotics tested. The high prevalence of multiple antibiotic-resistant organisms in the drinking water distribution system could potentially pose a threat to humans consuming this water. The presence of MAR organisms in the drinking water of Mafikeng, South Africa, is an important health concern due to the risk of developing waterborne diseases and the health risks associated with immunocompromised patients living in the area. It is therefore imperative to monitor the quality of water and strict quality control measures should be put in place to ensure the effective treatment of drinking water. Since the Modimola dam is the recipient of treated waste water as well as the water source for drinking water production, extremely strict measures should guide the waste water treatment plant. The quality of effluent leaving in this plant should be extremely high. This would decrease the load of microorganisms (and other contaminants) allowed to enter the dam. Such measures will improve the overall quality of water available for drinking water production, preventing outbreaks and spreading water borne diseases. Antibiotic resistance surveillance can be used as tool to control the problem of antibiotic resistance and to educate the public on the consequences of the misuse of antibiotics and also to regulate the usage of drugs in both human and veterinary medicine. It is also helpful to formulate guidelines for the optimal use of antibiotics. Further studies should be conducted to assess the level of antibiotics in water and the potential risks associated with human consumption of polluted water. It is also very important that findings from studies such as this one should be disseminated to the relevant stakeholders and the affected communities.

## Figures and Tables

**Table 1:** Oligonucleotide primers that were used for specific detection of *Pseudomonas* species.

Primer	Oligonucleotide sequence (5′-3′)	Target and size (pb)	PCR cycling conditions
ECF1 ECF2	ATGGATGAGCGCTTCCGTG TCATCCTTGCCTCCCTG	*ecfX, *(528)	35X 94°C for 45 s 58°C for45 s 72°C for 1 m

GyrPA-398 GyrPA-620	CCTGACCATCCGTCGCCACAAC CGCAGCAGGATGCCGACGCC	gyrB, (222)	35X 94°C for 45 s 66°C for45 s 72°C for 1 m

ETA1 ETA2	GACAACGCCCTCAGCATCACCAGC CGCTGGCCCATTCGCTCCAGCGCT	*toxA, *(367)	35X 94°C for 45 s 66°C for45 s 72°C for 1 m

Initial denaturing step of 95°C for 5 min and final strand extension of 72°C for 5 min.

**Table 2:** Oligonucleotide primers that were used to detect virulence genes in *Aeromonas* species.

Genes	Oligonucleotide sequence (5′-3′)	Target gene and size (bp)	PCR cycling conditions
*aer A *	Aer 2F: AGCGGCAGAGCCCGTCTATCCA Aer 2R: AGTTGGTGGCGGTGTCGTAGCG	*aerA* (416)	30X 95°C for 2 m 55°C for 1 m 72°C for 1 m

*hyl H *	Hyl 2F: GGCCCGTGGCCCGAAGATGCAGG Hyl 2R: CAGTCCCACCCACTTC	*hylH* (597)	30X 95°C for 2 m 55°C for 1 m 72°C for 1 m

Initial denaturing step of 95°C for 5 min and final strand extension of 72°C for 7 min.

**Table 3:** Average number of microorganisms isolated.

Site	Seasons	FC	TC	H	Ae	Ps
Molopo eye (ME)	Summer	30	40	>100	42	>100
	Winter	50	40	5	28	>100

Modimola dam (MD)	Summer	>100	>100	>100	>100	>100
	Winter	35	60	20	6	>100

Treated water ME	Summer	15	0	17	0	0
	Winter	0	0	7	0	0

Treated water MD	Summer	15	6	20	0	>100
	Winter	0	0	7	0	>100

Mixed water	Summer	5	5	10	0	0
	Winter	0	0	3	0	1

FC: faecal coliforms, TC: total coliforms, H: heterotrophic bacteria, and Ae: *Aeromonas*.

**Table 4:** Identification of the isolates from the treated (drinking) and source water using biochemical tests.

Site	API 20E (presumptive isolates)
Modimola dam (MD) untreated water	*Pseudomonas aeruginosa* *Pseudomonas luteola* *Aeromonas hydrophila* *E. coli* *Serratia odorifera* *Serratia liquefaciens* *Proteus vulgaris* *Providencia rettgeri1* *Chryseobacterium meningosepticum *

MD treated water	*Pseudomonas aeruginosa* *Pseudomonas luteola* *Serratia odorifera* *E. coli *

Molopo eye (ME) untreated water	*Serratia odorifera* *Serratia liquefaciens * *Aeromonas salmonicida *spp. *Pseudomonas oleovorans * *Chryseobacterium meningosepticum* *Salmonella choleraesuis* *Enterobacter asburiae *

ME treated water	*Serratia liquefaciens* *Myroides *spp.

Mixed water	*Serratia liquefaciens* *Serratia odorifera* *Aeromonas salmonicida *spp. *Pseudomonas aeruginosa* *Myroides *spp.

**Table 5:** Percentage of total coliforms resistant to various antibiotics.

Antibiotics	KF	AP	C	E	OT	K	TM	S	A	NE	CIP
Molopo eye *N* = 5	60	60	60	100	80	40	80	0	60	0	0
Modimola dam *N* = 5	40	60	20	100	40	20	80	0	80	20	0
Treated Molopo eye *N* = 1	100	100	100	100	100	100	100	0	100	0	0
Treated dam *N* = 2	100	100	50	100	100	50	100	0	50	0	0
Mixed water *N* = 2	100	100	100	100	50	50	100	0	100	0	0

**Table 6:** Percentage of faecal coliforms resistant to various antibiotics.

Antibiotics	KF	AP	C	E	OT	K	TM	S	A	NE	CIP
Molopo eye *N* = 5	40	60	0	100	40	20	80	0	60	0	0
Modimola dam *N* = 4	50	50	25	50	50	25	50	0	100	0	0

**Table 7:** Percentage of heterotrophic bacteria resistant to various antibiotics.

Antibiotics	KF	AP	C	E	OT	K	TM	S	A	NE	CIP
Molopo eye *N* = 5	80	60	40	80	20	40	80	0	80	0	0
Modimola dam *N* = 5	60	80	20	20	0	0	80	0	60	0	0
Treated Molopo eye *N* = 4	50	50	0	0	50	25	50	0	50	0	0
Treated dam *N* = 3	66.7	66.7	66.7	33.3	66.7	0	100	0	66.7	0	0
Mixed water *N* = 2	50	50	0	0	50	0	100	0	50	0	0

**Table 8:** Percentage of *Aeromonas* spp. resistant to various antibiotics.

Antibiotics	KF	AP	C	E	OT	K	TM	S	A	NE	CIP
Molopo eye *N* = 4	25	25	25	100	50	0	50	0	75	0	0
Modimola dam *N* = 2	50	100	50	100	100	50	100	50	100	50	0

KF: cephalothin, AP: ampicillin, C: chloramphenicol, E: erythromycin, OT: oxytetracycline, K: kanamycin, TM: trimethoprim, S: streptomycin, A: amoxicillin, NE: neomycin, and CIP: ciprofloxacin.

**Table 9:** Prevalent antibiotic resistance phenotypes observed amongst the bacterial groups.

Bacterial group	Antibiotic resistance phenotype
Faecal coliform	KF-AP-C-E-OT-K-TM-A
Total coliform	KF-AP-C-E-OT-K-TM-A
Heterotrophic bacteria	KF-AP-C-E-OT-K-TM-A
*Aeromonas* sp.	KF-AP-C-E-OT-K-TM-S-A-NE
*Pseudomonas* sp.	KF-AP-C-E-OT-K-TM-A

KF: cephalothin, AP: ampicillin, C: chloramphenicol, E: erythromycin, OT: oxytetracycline, K: kanamycin, TM: trimethoprim, S: streptomycin, A: amoxicillin, and NE: neomycin.

**Table 10:** Number of heterotrophic bacteria (H), *Pseudomonas* (PS), and *Aeromonas* (AE) isolated from different sites within the various clusters.

Site	Cluster 1 (Number of isolates = 21)			Cluster 2 (Number of isolates = 8)		
	H	AE	PS	H	AE	PS
Dam	5	2	0	0	1	1
Eye	4	2	0	1	1	1
Treated water dam	2	0	0	1	0	1
Treated water eye	3	0	0	0	0	0
Mixed water	2	0	0	0	0	1
